# Concept of Educational Assistance to Health Protection of the Individual

**DOI:** 10.5539/gjhs.v8n3p122

**Published:** 2015-07-13

**Authors:** Elena Aleksandrovna Levanova, Olga Rafailovna Kokorina, Yuriy Vladimirovich Nikitin, Tatiyna Vladislavovna Perepelkina, Polina Anatolievna Segodina

**Affiliations:** 1 Moscow state pedagogical university, M. Pirogovskaya str., 1, Moscow, Russia; 2 Sakhalin state university, Lenin str., 290, Yuzhno-Sakhalinsk, Russia

**Keywords:** concept, educational assistance, health-protecting technologies, health protection, individual, health, healthy lifestyle, professional education

## Abstract

The article describes the theoretical and practical need for the development of the concept of assistance to health protection of the individual in order to address the problem of health protection of students and teachers in the conditions of a higher pedagogical education. The problem of studying human health, its entirety, systemacity and connection with the environment attracts particular attention in recent years. This was one of the reasons to study the problem of “healthy lifestyle” as the qualitative characteristic of a human life aimed at health, due to the fact that a healthy lifestyle is one of the determinants of health. This is made possible with the use of specific health-protecting technologies aimed at searching for ways and means of protection and conservation of health of students and teachers in the conditions of the educational process and using educational tools, which is currently included into the priorities of education.

## 1. Introduction

The strategic aims of education outlined in the “National Doctrine of Education in the Russian Federation through to 2025” are closely linked with the problems of Russian society, including “the creation of a framework for sustainable social, economic and intellectual development of Russia, ensuring a high quality of life of the people and national security”. Main goals and objectives of education in the Doctrine include “fostering healthy lifestyle, development of children and youth sports”, and the main objectives of the state in education include a comprehensive care for “the protection of life, health and physical education in the development of children, pupils and students”.

Scientific and practical need to develop special health-protecting technologies, which mobilize resource potential of the participants in the educational process to achieve the objectives of the new educational paradigm, is increasing. In this regard, assistance to the protection of health as a process aimed at self-regulation of an individual and finding the meaning of a healthy lifestyle becomes the strategy of development of health-protecting technologies in an educational institution (Serikov, 2002).

Analysis of the practice of educational institutions revealed the importance of assistance to health protection in the following aspects: relevance of subjects of educational process that have health protection competence; need for technological support of the formation of the axiological attitude to health as a factor of personal development and its implementation in the process of interaction between the subjects of the educational process (Balsevich, 1990).

Currently, there are significant, unreasonably high overloads of the participants in the educational process in the system of basic and vocational education in Russia. Mechanisms of self-regulation of the individual begin to function at extreme values of life potentials of human health, creating a threat of its deterioration. Fatigue affects the health of participants in the educational process (Pasyukov & Kokorina, 2003). In addition, too severe regional climatic conditions that are characteristic of, for example, the Far North and equivalent areas divert the natural forces of the body to opposing them in terms of physical survival. This can also be a threat to health safety of the participants in educational process (Pasyukov, 2004).

There is a social and educational need to study the values of health protection as a strategic part of educational activities of the educational institution that seeks not only to fully satisfy, but also create a need for a healthy lifestyle in the context of axiology of the health protection.

With all the real-life opportunities for educational assistance to health protection of the individual, today its potential as a pedagogical phenomenon is poorly understood and therefore not realized. Thus, the need arises for theoretical basis for constructing educational concept of assistance to health protection of the individual in terms of higher pedagogical education.

The emergence of the problem and the relevance of its research are determined by aggravation in educational theory and practice of a number of contradictions between:

a) society's need for a high level of health of the younger generation as a condition for the further development of the society and the lack of effectiveness of existing educational concepts and pedagogical tools aimed at protecting and improving the health of participants in the educational process;

b) the increasing demand for scientific (educational) feasibility of health protection of the individual in the conditions of higher pedagogical education and the lack of a holistic concept of self-educational activity of students of the higher education institution;

c) the ever-increasing potential of educational technologies aimed at optimizing the health protection of the individual and the lack of their relevance in the system of higher pedagogical education;

d) objective requirement to have a healthy lifestyle to ensure the health of the individual and insufficient development of its pedagogical foundations: the content of educational technologies that ensure its effectiveness in the conditions of higher pedagogical education.

## 2. Methodology

Given these theoretical positions, the experimental part of the study was conceived and implemented as experience of the development and empirical validation of the conceptual model to assist health protection of the individual in the conditions of pedagogical education, in order to determine the conditions and factors of development and integration of the structural components, their systematic interconnection and interdependent aggregate.

The following tasks have been put forward in the pedagogical experiment:

- practical implementation of the concept of educational assistance to health protection of the individual;

- experimental verification of pedagogical conditions of the concept;

- experimental approbation of the developed stages of the study;

- determination of the effectiveness of the introduction of the concept of education assistance to health protection of the individual.

Experimental work was carried out in three stages.

During the first stage (preliminary stating), the organization of educational process in higher education institution has been comprehensively studied, and the initial state of the health-protecting activities was defined; selection of specific techniques for the study of the initial state of the experimental object; definition of the features by which we can judge about the changes in the experimental object under the influence of appropriate educational assistance.

During the second stage (substantive and procedural), the formative experiment was conducted, during which the probation was determined: selection of the content of the experimental work; carrying out the experiment on determination and validation of pedagogical conditions; formation of the experimental method performance criteria for health-protecting training of students; recording of data on the course of the experiment based on the intermediate sections describing the object changes under the influence of the experimental system of measures.

The third stage (control and adjustment) included: analysis of the obtained experimental data; verification of the analytical material with the purpose, objectives and hypothesis of the study; processing of the results of the experiment, control diagnostics of level of assistance to health protection of the individual; description of the features of the subjects of the experimental treatment (students and teachers); comprehension and analytical presentation of the material and findings.

To achieve the objectives and verify the initial assumptions, set of the research methods was used: methods of theoretical analysis (historiographical, retrospective); prognostic methods (modeling the educational process, summarizing the independent characteristics, expert assessments); diagnostic methods (questionnaires, interviews, discussions, evaluation, ranking, tests); archival methods (analysis of the products of work of teachers and students: creative individual assignments, essays, term papers and dissertations of students, reflection of the results of diagnostic and self-diagnostic); observational methods (direct and indirect observation); study and generalization of mass and excellence experience; experimental work; methods of statistical data processing (Guilford, 1991).

## 3. Results

Adoption of healthy lifestyle of young people in general and students in particular is regarded today as one of the priorities of the development of Russian education system. Significance of the formation of values of a healthy lifestyle is due to the need to maintain and increase the health of students and improve their physical, mental and social wellbeing. Good health and physical performance are a condition and a basis for unlocking the creativity potential of the individual, their professional self-realization (Kokorina, 2008).

The main goal of health-protecting education is to equip a person with scientific and theoretical knowledge about the formation, preservation and strengthening of health and practical knowledge of healing the body.

Understanding the philosophical, psychological and educational nature of the assistance to health protection of the individual in the conditions of the pedagogical education makes it possible to consider this process as a pedagogical concept, which defines the methodological approaches to assist the health protection of the individual in the conditions of the pedagogical education; main pedagogical trends and principles, a set of pedagogical conditions that increase the effectiveness of the assistance to health protection of the individual.

Study of methodological approaches, as well as the analysis of existing practices, allowed to define the concept of assistance to health protection of the individual in the conditions of the pedagogical education as a system of modern scientific and pedagogical views, ideas, target settings, priority areas, forms, methods of joint activity of participants in the educational process in forming a healthy lifestyle, readiness for professional health-protecting activity (Belikov, 2004).

Methodological basis of our concept is the methodology of the systematic, pragmatist, person-centered and axiological approaches to the study of nature and mechanism of interaction between the individual and the environment, the role of a healthy lifestyle in the development of the individual (Aizman, 2008), as well as the link between the qualities of a human as an individual and the same qualities as a subject of educational activities, philosophical, social, psychological and pedagogical theories, ideas, concepts providing an understanding of various aspects of health protection of the individual and realization of the implementation of assistance to health protection in the educational process of the high school.

The content of the pedagogical concept is defined by its key provisions that make up the original theoretical basis or a core–a system of initial assumptions that determine the features of construction of scientific theory and characterize its specificity. Based on this understanding, the core of the pedagogical concept should include laws and principles of assistance to health protection of the individual that allow to explain its nature and ensure the ability of theoretical and logic conclusion of all provisions of the pedagogical concept (Yakovlev & Yakovleva, 2006).

The main regularities in the study are identified as follows: effectiveness of assistance to health protection of the individual in the conditions of the pedagogical education is determined by taking into account dependencies such as the dynamics of the psycho-emotional state and health of students and the subsequent development and use of programs of correction of these states on the basis of the organization of humanistic interaction between students and teachers and the support of appropriate pedagogical principles.

Implementation of the above regularities is based on the following principles of humanistic pedagogy that determine educational assistance to health protection of the individual in the system of pedagogical education: the principle of the integrity of the individual, their psychosomatic, social and cultural unity, integrative exposure to health-protecting activities. At that, the priority components in the process of health-protecting activities should be self-realization, self-development and creativity that are in harmony with the inner nature of a human; principle of motivation of students to self-knowledge and self-actualization as unlocking their self, inner activity, initiative, where the teacher plays the role of an assistant, has found a certain reflection in the theoretical and practical exercises of Komensky, Pestalozzi, Diesterweg, Tolstoy, Ushinsky, Sukhomlinsky; the principle of joint action, based on the communicative exchange, enrichment of the student's personality, acquiring the experience of communication – according to the strategy of cooperation, the identity of the student is in the center of education, the purpose of education should be in harmony with both the purpose of life of the individual and society; principle of health protection of the individual in the educational process involves the purposeful combination of energy-intensive labor of the education participants with energy savings of each of them, which is achieved through the creation of knowledge and the needs of a healthy lifestyle.

Fundamentally important position in the concept is that both teachers and students are a subjective basis. This means that the goal of assistance to health protection of the individual can be achieved at the simultaneous protection and formation of health of the educators and students (Pasyukov et al., 2006).

Theory and practice of pedagogical education rightly asserts that any pedagogical problem can only be solved with the help of adequate technology implemented by a qualified professional educator.

“Educational technology is an ordered and task-structured set of activities, operations and procedures to ensure correctly measured result in a changing environment” (Levanova et al., 2008). This definition most accurately reflects the dynamic process of educational assistance to health protection of the individual in the system of the higher pedagogical education.

In implementing the concept of assistance to health protection of the individual, we adhere to the following phases of educational technology: organization of the process; diagnostics; content of the implementation process; evaluation and analysis of results.

In the context of assistance to health protection of the individual, teachers and students communicate on the basis of “partnership, collaboration and cooperation”. Such activities form the norms of cooperation, adjust behaviors, ways of interacting, which subsequently, in other educational systems (school) will express as acts of health protection. Communicative and technological skills are a form of intellectual and practical knowledge, and, consequently, their acquisition is related not only to the methodological aspects, but also to didactic (Pasyukov, 1999).

In the course of experimental work in the experiment was attended by 976 people, including 469 of 507 students and teachers. To ensure reliability and reproducibility of the research results the experimental work was conducted in four streams, repeated in different conditions and with different co-stav members.

Ascertaining stage of the experiment showed that the students of experimental and control groups was approximately at the same level of motivation of choice of profession, health status, readiness to the health improvement activities. At the end of the forming experiment in the control phase we conducted (according to the same method as that used for summative stage) final measurements of the level of adaptation of students to the conditions of professional education on the list of leading pedagogically driven ka quality.

Without changes in the control group remained an indicator of being able to organize their training activities, whereas students in the experimental group during the experiment, the figure took I took 1,2 times. This is due to the implementation of the pilot program of activity of the College for creating an environment of students ’adaptation to the conditions of professional education, transition to teaching in the “College-University” overall, which ranks independent work of students.

The observed increase in the values of the rate of formation of the desire to get a profession span of dagogo and work in this area the students of the experimental group in 1,5 times in comparison with the outcome strength indicator that indicates, in General, positive adaptation to the conditions of the these same indicators in the control group, the number of students showing sustained a genuine interest to the profession, increased only 1,7 times.

To determine the dynamics of relations second-year students to the conditions of professional training during the pedagogical experiment was conducted a second survey, which displays ruzhil: if in the first year leading motive of learning the teaching profession was the interest to a subject, in the second year of such a motive was the opportunity to train and educate children (68,9 per cent), i.e. there is a change in orientation of the individual towards teaching. Because of the changes undergone other motives for learning.

On indicators of development of academic skills in the course of the experiment was noted chen positive dynamics in the formation of such skills as the ability to write lectures, organized to activity and the ability to analyze the results. So the success of educational activity of students of the experimental group indicates increase academic achievement in core subjects, which was measured by the average score. So in the first year average score of students was 3,6, while the students of the second course – 4,2 points. Similar performance of the students in the control group was composed of respectively 3,7 and 3,9 points.

Experimental work in these areas showed the effectiveness of pedagogical co-operation in the educational process of students in the experimental group, which is confirmed by the positive dynamics of the level of adaptability of the individual.

It was observed a decrease in the level of trait anxiety of the students of the experimental group. This facilitated the development of interpersonal communication in the process of joint activity of students and teachers, development of communicative skills in the study of psychological and pedagogical disciplines. The level of trait anxiety of the students of the experimental group, the reduction declined by 1,4 times, which is an indicator of adaptability of the individual to the learning environment, while students in the control group the decrease in personal anxiety occurred slightly and this collection rate at the time of the study continued to remain close to high.

The criteria for evaluating the effectiveness of promoting the health of students to the conditions of professional education in the experimental group were marked differences in the health status of students at different stages of adaptation. So, if in the initial stages of adaptation is a decrease in the level of physical health of freshmen, the analysis condition before the health of the students of the second course gives grounds to draw a conclusion about the positive dynamics of the studied parameters. At the same time, in terms of health index, an increase in the number of second year students of experimental group who did not apply to the doctor during the experiment, whereas in the control group among the students - sophomores an increase in this indicator slightly.

The reasons for the poor health status of students of the control group in contrast to the experimental, in our opinion, was the lack of teaching of health saving space, which is one of the conditions for promoting a healthy lifestyle students tov in the period of adaptation to the conditions of professional education.

Positive dynamics of health status of students of the experimental group comes amid increasing their awareness in the field of healthy lifestyles, the growing use of the developed tools and methods of health preservation in the educational process. This helps the students as quickly as possible to “slip” the initial phase of adaptation, allows to obtain the profession of teach-the determinant with the lowest energy demands (physical, mental, spiritual).

To determine the dynamics in orthobiosis students, we conducted repeated measurements and compared indicators obtained on the basis of the same methodologies as in the control phase.

Speculative enough students actively recognize all 13 factors orthobiosis and recognition is higher among students of the experimental group is 80,6% (compared to control group 74,2 per cent). Increase in perfect view of it from the students of the experimental group was 7,1%. The students of the control group and 1,3%. Real respect for students orthobiosis continues to be below the ideal representation of it (the experimental group -66,8%, control 52,3%). The increase in the performance of the experimental group were significant and amounted to 15,7%, in control group the increase was only 1,5%. Students realized the need to take care of their health and in the experimental group the increase in this factor was 20%, rationally organized his nutrition and sleep – an increase of 12%. Increased performance for the factors of “self-control” and “optimistic mood”. Increase, respectively, 16% and 32%.

These results indicate that students recognized the role of self-control and optimization to statistical sentiment to preserve and strengthen their health. Much has changed for the better indicators for the factor “avoiding alcohol and quitting Smoking. Quit Smoking and/or throw 36% and restricted the use of alcohol 12%. Significant dynamics of spacecraft in the indicators for the factor “dedication to work/school” – 36%. These students appreciated the role of commitment in work/school as the basis of optimism, professional with insolvency and background to creative self. In the control group also noted changes in the real observance of orthobiosis, but the trend is insignificant. The increase occurred for the factor “observance sleep” 8% “optimistic mood” 4% “abstinence from alcohol” 4% and “dedication to work/school 4%. Unfortunately increased by one the number of Smoking students in con-control group. This is all the more negative as students majoring pedagogy and methods of primary and pre-school education are mostly girls. The growth indicators in the control group, we can explain the fact that students learn the discipline of the natural Sciences, psychology and pedagogy, as well as the course “human Ecology”, which get some knowledge in the field of healthy lifestyle.

Factor analysis at the final stage of the research allowed us to determine the levels SFOR-valuations of orthobiosis students of both groups in the dynamics.

The number of students with a high level of orthobiosis in the experimental group increased by 24%, with the average level decreased by 4%, with a low level cut-elk 20%. In the control group, the situation has changed slightly: the number of students with a high level of orthobiosis increased by 4%, the number of students with a medium level of orthobiosis decreased by 4% and the number of students with a low level of orthobiosis remained unchanged.

The obtained results allow to conclude that students are prepared for a healthy moviesparade activities in the school system.

To determine the change in the value of students ’attitudes towards their health, we conducted repeated measurements and compared the performance obtained using the same techniques.

The data showed the dynamics of awareness of the value of health during the experiment. The growth of health indicators, placed first in a number of securities of power the students of the experimental group was +60%. The students of the control group it was only +12%. Positive growth rates in the pilot Noah group allows us to conclude that the training of students of pedagogical higher education institution to the health improvement activities in the school system is successful.

The measurement results of the final experiment (control) phase of study by means of drawing up the structural systemic criterion showed that the experimental group on these indicators is far ahead of the control group. Positive pedagogical effect is achieved. The effectiveness of the promotion of health protection of the person in 1,6 times higher.

The findings suggest that the experimental work resulted in creation of the effective system to assist health protection of the individual in the conditions of higher pedagogical education implemented in the period of study at the higher educational institution, in the course of professional activities. It was based on the developed and tested conceptual model, and the proposed health-protecting technologies allowed to ensure the unity and interrelation of units of this model to assist health protection of the individual in the conditions of higher pedagogical education.

## 4. Discussion

Problems of our study on the substantive and procedural levels are solved by the implementation of the identified set of principles of assistance to health protection of the individual in the conditions of higher pedagogical education, which are:

- scientific principle that defines the scientific knowledge as knowledge of basic ideas, concepts, laws, regularities of assistance to health protection of the individual, knowledge of leading pedagogical theories, basic categories and concepts of health-protecting pedagogy, mastery of systemic knowledge about the laws of human's connection with nature and society, processes of formation of a healthy personality in the real socio-cultural and educational space; development of creative potential of each teacher, formation of internal setting for self-education, self-education and self-development, formation of scientific knowledge about health through the development of set of disciplines aimed at student mastering theoretical and methodological foundations necessary for teachers in the future health-protecting activity (Smirnov, 2005);

- systemacity principle that considers the theory, system of concepts, trends, contradictions, laws and regularities in the relationship and interdependence, dynamics of development of all components of assistance to health protection of the individual, depending on the effectiveness of their implementation at every level; content of the professional activity of the teacher aimed at health protection in accordance with the objectives of pedagogical education that involve continuous social and moral common cultural and professional development and ability to choose pedagogical tools that enable the development of motivational value systems to strengthen and protect health;

- regionalization principle considering social and economic conditions of the region, climatic and geographical conditions, local and national peculiarities of functioning not only of the institutions of pedagogic education, but also the functioning of the regional educational system as a whole; according to some scientists, this principle emphasizes the tying of conditions in which a human develops to the place of residence, population in the geographical, social, economic terms (Nesterov, 1998);

- variability principle suggesting at various levels of assistance to health protection the provision of substantial, pragmatist and organizational abilities to meet the professional and personal needs;

- principle of focus of the educational process on the subject development and self-development, involving the design and implementation of training programs and health-protecting technologies contributing to the development of mechanisms to facilitate assistance to health protection of the individual;

- adaptability principle: manifestations of the individual's health depend on the specific adaptive situation, in this regard, this principle contributes to the ability of the individual to adapt to the conditions of professional education through the creation of an environment for the adaptation of the individual in the educational space of the higher education institution, which is one of the ways to assist health protection, because the human health is based on adaptive mechanisms (Melinda et al., 1993);

- pedagogical appropriateness that allows students to differentiate values at the level of analytical, evaluative and predictive skills, build pedagogically reasonable relationships, regulate the inside and outside team relationships, and predict the outcome of interaction;

Implementation of these principles, justification of the essence and content of assistance to health protection of the individual in the conditions of pedagogical education, analysis of the basic contradictions in the educational environment, variety of positions of the modern paradigm of education and modern concepts of assistance to health protection of the individual allowed to establish dependence manifested in the form of the leading trends.

We single out a humanistic focus of health-protecting education as one of the leading trends in assistance to health protection of the individual in the conditions of pedagogical education.

Humanization of pedagogical education is inextricably linked with the creation of conditions for creative self-identity, choice of the area of activity, circle of meaningful communication and place of force. It is this area of human relationships in an educational institution that is today an area of anxiety and concern, because it is largely determined by the measure of human maturity of the graduate and their professionalism (Kulikova, 2001).

Humanization is one of the priorities of innovation in the field of multi-level professional education. This problem has always been the focus of scientists-philosophers, sociologists, psychologists and educators.

Humanization of vocational education provides the proper formation of the humanistic in a human. Teach something and make socially useful are technical means for the main function of humanization. It is important that an individual has found their identity and could realize it. It is important what image of an individual develops and how it is practically embodied in their life interval. It is an extremely global function of humanization of professional education (Nain, 2003).

Therefore, it is important to establish the humanistic nature of interaction between the participants in the educational process.

Interaction can be either formal, defined by the scope of “teacher - student” and the respective chain of command, or have a nature of substantial, creative union, where a teacher and a student become colleagues, peers, advancing in their self-development. Humanistic character of interaction between the participants in the educational process is an important condition for maintaining their mental health. As is well known, physical and mental health are interrelated and interdependent.

The trend of value-based health protection of the individual. Based on these traits of the individual, in our view, creation of the environment for students to adapt to the conditions of professional education will be successful with the implementation of professional focus of the content and organization of the educational process, and when bearing in mind that any kind of human activity, including skills development, is based on physiological phenomenon, the development of frameworks and norms of a healthy lifestyle becomes necessary in order to protect and strengthen the health of the individual. Implementation of the factor of health-protecting focus of the individual becomes possible with the creation of the educational health-protecting space.

Students’ knowledge of means and methods of forming a healthy lifestyle and ways to improve and maintain their health is required for the formation and integrated development of pedagogical health-protecting space in pedagogical education institution. This work includes the development of special training programs (blocks, modules) to inform students about the rules and regulations of a healthy lifestyle.

In accordance with the standard of secondary and higher professional education, students study such subjects in the block of disciplines of biomedical training in the first year of pedagogical college as “Developmental anatomy, physiology and school hygiene” and “Basics of a healthy lifestyle”. Their goal is to form a representation by students of children's bodies and their functional features in different ages, of the hygienic requirements for the educational process, and of the basics of a healthy lifestyle factors. However, these disciplines are not focused on the personality of the student as a future teacher, they do not contain any specific recommendations for health protection in the process of teaching and professional activities.

In this regard, the development of the problem of protecting and strengthening the health of students is of paramount importance for professional schools not only in theory but also in practical terms: the establishment of a harmonic connection between education and health provides quantitative and qualitative changes in the development of the student. In this work, the unity of action of pedagogy and hygiene is important (Anderson, 1984).

The trend of technologizing of the assistance to health protection of the individual in the conditions of pedagogical education is a leading trend of formation of readiness of the future teachers to use health-protecting technologies in practice.

Our research and experimental work was focused on finding health-protecting technologies as a means of assistance to health protection of the individual in the context of a holistic pedagogical process based on the indissoluble unity of theoretical and practical training, various directions of teaching activities. We consider the educational process in the logic of the main components of practical readiness and stages of teaching. So, at the first stage – adaptation – the target setting for interaction based on the proposed meanings and values of the upcoming activities involves solving problems of formation of motivational and axiological attitude to joint work, creating emotional state for acquisition of specific practical skills that will help in mastering the profession, establishing contact, overcoming the stereotype of the object-subject relations, establishing a positive psychological climate.

The specified trends and principles are logically interrelated. Their implementation determines the psycho-pedagogical conditions of the effective assistance to health protection of the individual.

Such conditions are: creation of the environment for adaptation of the individual in the educational space of the higher education institution; establishment of humanistic interaction between the participants of the educational process in the joint curricular and extra-curricular activities; creation of pedagogical health-protecting space for maximum efficiency of physiological, psychological functions and behavioral reactions of the individual in the educational process; provision of adequate training of pedagogical staff to organize health protection of the individual.

## 5. Conclusion

The concept of assistance to health protection of the individual can be effectively implemented in the system of pedagogical education at the high organizational culture of the pedagogical education institution, qualification of the teacher (teaching staff) in this work, sufficient scientific and methodological support and application of performance criteria to activities of the students that are adequate to the task.

Educational technology of assistance to health protection of the individual in pedagogical education is aimed at consistent and continuous movement of interconnected components and stages, states of the educational process and actions of its participants. Each stage of the health-protecting activity corresponds to its own objectives, content, methods and results.

The developed conceptual framework of health protection of the individual in the conditions of higher pedagogical education opens a new direction of scientific and pedagogical research, which requires solving a number of undeveloped significant aspects of this problem. The promising direction of its solution is systematization of methods and technologies of health protection; development and testing of tools to optimize health-protecting environment in the context of higher pedagogical education. Scientific development of these and other problems in the pedagogical aspect will contribute to health protection of future teachers in the process of study at the higher education institution.

Solution of this problem should ensure not only a healthy lifestyle, comfort in the classrooms, teaching students skills to outline lectures and organize independent work, widespread introduction of computer technology, balanced diet, optimal mode of work and rest, treatment and preventive care, medical examinations, etc. but also the proper organization of the learning process.

This requires the researching teachers to develop appropriate recommendations aimed at strengthening and maintaining the health of students of pedagogical vocational education institutions, and as a consequence, improvement of the quality of education. Convergence of training and education about hygiene and prevention improves health and health promotion students. This is possible by combining the efforts of the team of the vocational education institutions and health care. Developing a holistic approach to the problem of health of students is directly related not only to the requirements of today, but also to the prospects of development of vocational education system, which in turn causes the consideration of it as a systematic and holistic education that models the activity and behavior of a human in the conceptual space of the subject-subject and subject-object relations.
